# Oro-Maxillofacial Radiology and Imaging: An Update

**DOI:** 10.2174/1874210601711010334

**Published:** 2017-06-30

**Authors:** Deepak Gupta (Guest Editor)

**Affiliations:** Reader, Department of Oral Medicine and Radiology, M.M. College of Dental Sciences and Research, Mullana, Ambala, Haryana, India Tel: +91-9896671281; E-mail: drdeepak_26@rediffmail.com

It is a well known fact that conventional Dental radiographs were considered as an important part of dental care in the earlier times. Most of these radiographs are still practiced widely by a lot of dental practitioners. In the past, the field of "Dentistry" was precise and was considered related to dentition only. Along with clinical oral examination, the radiographs used to provide with a more complete view of the tooth and its associated structures. Now a days, Dentistry has elaborated its scope. These days the dental professionals are involved in the diagnosis and treatment of diseases and disorders of not only the Oral cavity but the maxillofacial region as well. Now a days, the concept of conventional radiology is overtaken by "Digitalized Radiology" which has further aided the dental professionals in diagnosis and treatment planning along with reducing the radiation exposure of the patient.

As was discussed in the last thematic issue, fast technologic evolution in the field of maxillofacial radiology has made it possible to make utmost accurate and safe diagnosis. It aids in identifying diseases and developmental problems before they become serious health issues. This has further led to a precise treatment planning as well as follow up.

Although the dental radiology is considered as a back bone of dentistry, there is a need to justify the radiographic examination performed whether it is an IOPA (Intra-Oral periapical radiograph) or a CBCT (Cone Beam Computed Tomography). It is attributed to the fact that the patient is exposed to X-radiation which is harmful for the patient. However, the amount of radiation used to obtain dental radiographs is very small; the dentists must follow the ALARA principle, which stands for “As Low as Reasonably Achievable,” when obtaining radiographs.

The dental professionals must decide when radiographs are needed on the basis of oral examination findings, symptoms reported or on the basis of review of health history or risk of experiencing oral disease of the patient. Radiation safety principles must also be followed so as to cut down unwanted radiation exposure which includes the usage of the fastest image receptor; reduction in the size of the x-ray beam to the size of the image receptor whenever possible; use of proper exposure and processing techniques; use of leaded aprons and thyroid collars. Further, the dental professional must always consider utilizing the older radiographs performed by other dental professionals whenever possible. This also will help to limit patient’s exposure to radiation.

Owing to the unmatched importance of maxillofacial radiology, this particular Guest Edited issue is not only dedicated to update the current research and knowledge regarding the role of maxillofacial radiology in the diagnosis but to make the dental professional aware of the radiation hazards as well so as to minimize the radiation exposure. Henceforth, I would like to congratulate the vision of the editorial board of *The Open Dentistry Journal* at Bentham *OPEN*, for realizing a special issue on this topic. This issue has included original peer-reviewed contributions inclined towards the diagnostic oral and maxillofacial radiology and related imaging sciences. Below is a brief description of the articles of the special issue.

1. Comparison of Mesiodistal Root Angulation Measured from Conventional and CBCT Derived Panoramic Radiographs.

This particular article highlights the use of cone beam computed tomography (CBCT) in orthodontics. The objective of this study was to manipulate CBCT panoramic reconstruction to make it comparable to conventional panoramic image and to compare mesiodistal root angulations on both images. Concurrent conventional panoramics and CBCT volumes were obtained from 40 subjects. CBCT volumes were manipulated to generate pan-like images that mimic the occlusal plane angle of the corresponding panoramic, allowing comparison of mesiodistal root angulations and determination of the head tilt required to produce the reconstruction. It was concluded that CBCT pan images must be used with caution due to variation between methods in specific areas of arches. The images can be useful for assessment of mesiodistal root angulations if the volume is properly manipulated to create a pan-like image.

2. Efficacy of Panoramic Radiography in Detection of Osteoporosis in Post-Menopausal Women When Compared to Dual Energy X-Ray Absorptiometry.

Since Osteoporosis affects many elderly people and might not be detected until symptoms of fractures occur, the aim of this study was to determine the validity of the Klemetti Index (KI) measured on panoramic radiographs, in the diagnosis of osteoporosis. Postmenopausal women were selected and a panoramic radiograph was taken to grade their mandibular cortex on the basis of Klemetti Index. Later, all the patients were subjected to dual energy X-ray absorptimetry scan for estimation of bone mineral density. It was concluded that panoramic radiographs are useful for screening of osteoporosis with the usage of Klemetti Index. It can serve as a subsidiary diagnostic tool in early detection as well as referring the suspected patients to bone densitometry clinics.

3. Evaluation of Distolingual Canal/Roots in Mandibular Molars and Mesiobuccal Canals in Maxillary Molars by CBCT

The aim of this study was to identify the bilateral distolingual (DL) canals / roots of the mandibular first molars and second mesiobuccal (MB2) canals of the maxillary first molars in the same Turkish individuals using cone-beam computed tomography (CBCT). A total of 150 CBCT images showing all mandibular and maxillary first molars were retrospectively investigated in a Turkish subpopulation. The patient’s age, sex and presence of roots and canals were assessed. The frequency of bilateral DL canals, DL roots, and MB2 canals were investigated. It was concluded that CBCT is an effective tool for the detection of additional distolingual canals/roots and second mesio buccal canals, and is a valuable aid for dentists performing root canal treatment.

4. Role of Sinonasal Anatomic Variations in the Development of Maxillary Sinusitis: A Cone Beam CT Analysis.

Since several anatomical variations can lead to the inflammation of the paranasal sinuses; therefore, surgeons should be familiar with these variations and their impacts on the status of the paranasal sinuses. Henceforth, the present study was aimed at determining the prevalence of Haller cells and its association with patients’ sex and age. Furthermore, the relationships between the presence and size of Haller cells, deviation of the uncinate process and size of the maxillary sinus ostium with the occurrence of maxillary sinusitis were investigated. It was found that there are statistically significant associations between both the presence and surface area of Haller cells and the occurrence of ipsilateral maxillary sinusitis. Neither the angulation of the uncinate process nor the size of the maxillary sinus ostium significantly correlates with the formation of maxillary sinusitis.

5. Facial Soft Tissue Thickness of Midline in an Iranian Sample: MRI Study

Different methods have been used to identify the human skeletal remains and important data can be obtained using these techniques too. However, facial reconstruction is the last method to indentify unknown human faces which require knowledge about facial soft tissue thickness in the different positions of the face. The present study determined the facial soft tissue thickness in the different landmark points on the MRI images of patients.

6. Computed Tomography in Craniofacial Fibrous Dysplasia: A Case Series with Review of Literature and Classification Update.

Fibrous dysplasia (FD) is an uncommon but important lesion affecting the maxillofacial region as it can cause severe deformity and asymmetry of face and jaw bones. Craniofacial fibrous dysplasia (CFD) is one of the types of FD that can affect the bones of the craniofacial complex, including the mandible and maxilla. It can be investigated by conventional radiography, computed tomography (CT), scintigraphy, and histopathology. It shows various radiographic findings according to the degree of maturation which determines the degree of opacification. Various appearances described on conventional radiographs are radiolucency, ground-glass, smoky, cloudy, peau d’orange, finger print, or diffuse sclerosis, however CT features has not been widely discussed. This article highlights the relative importance of computed tomography in the diagnosis and assessment of CFD as the margins, extent, internal structure and effect on surrounding structure are best detected on computed tomographic images.

7. Ultrasonographıc Appearances of Cervıcal Lymph Nodes in Healthy Turkısh Adults Subpopulatıon: Prelımınary Study

The purpose of this study was to investigate whether there was any association between age, gender, body mass index (BMI), nodal morphology and vascular pattern in healthy Turkish adults. It was concluded that normal cervical lymph nodes are oval, with an unsharp border and an echogenic hilum but no relation between the age, gender and BMI. Also ultrasonography is an applicable imaging modality for examination of cervical lymph nodes. 

8. Giant Complex Odontoma of Mandible: A Spectacular Case Report.

Odontomas are non aggressive, hamartomatous developmental malformations composed of mature tooth substances and may be compound or complex depending on the extent of variety of morphology or on their resemblance to normal teeth. Among them, complex odontomas are relatively rare tumors. They are usually asymptomatic. Occasionally, these tumors become large, causing bone expansion followed by facial asymmetry. Odontoma eruptions are uncommon, and thus far, very few cases of erupted complex odontomas have been reported in the literature. The aim of this paper was to report the case of an unusually large, painless, complex odontoma located in the left posterior mandible.

The above mentioned articles highlight that maxillofacial radiology is an integral part of dentistry and is being practiced widely. Further, there are documented facts that the knowledge of the general dental practitioner regarding the radiation hazards as well as the methods of minimizing radiation exposure along with apt diagnostic information is lacking. Sometimes diagnosis by conventional radiographs is equivalent to the higher imaging modalities and sometimes modalities like ultrasound and MRI can give better results as compared to the modalities which use ionizing radiations. Henceforth, this thematic issue offers a forum for international collaboration in diagnostic imaging of the dental and maxillofacial region, so as to raise awareness about the diagnostic application of various arms of maxillofacial radiology along with justification of the radiograph prescribed. The featured articles include cutting-edge research papers, a review article and a case report that will surely appeal to clinicians involved in the diagnostic radiology and imaging.

The editorial board of this special issue of *The Open Dentistry Journal* features a panel of international expert reviewers who has provided their expertise and guidance in shaping the contents of the issue. Once again I would like to extend my thanks to the Editor in Chief of *The Open Dentistry journal* for sponsoring this important special issue to uncover the current state of the art scientific literatures and future direction in maxillofacial radiology.

